# Supplementary material for: Blood and Salivary Inflammatory Biomarkers Profile in Patients with Chronic Kidney Disease and Periodontal Disease: A Systematic Review
Source: Diseases. 2022 Feb 17;10(1):12. doi: 10.3390/diseases10010012 (PMC8883939; doi:10.3390/diseases10010012)
Supplement: Supplementary file 1 [file diseases-10-00012-s001.zip › diseases-1573382-SI.pdf]

Supplementary Materials

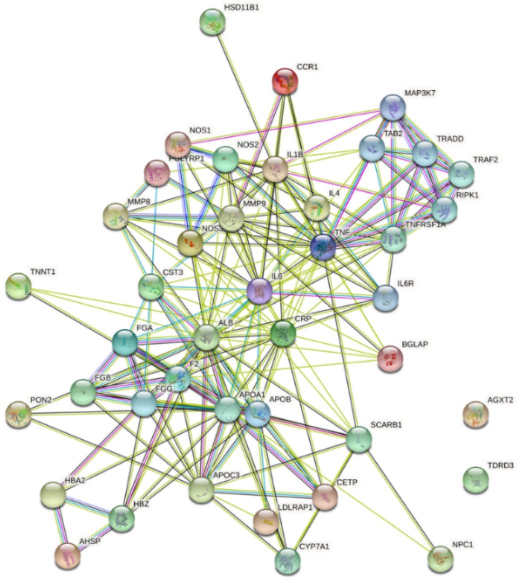

Supplementary Figure S1. Proteins found in the included studies (cross-link).

Supplementary Table S1. Details about the proteins and other biological indicators used as biomarkers in the included studies.

**CRP**	C-reactive protein: Displays several functions associated with host defense: it promotes agglutination, bacterial capsular swelling, phagocytosis, and complement fixation through its calcium-dependent binding to phosphorylcholine. Capable of interacting with DNA and histones, and may scavenge nuclear material released from damaged circulating cells.
**Fibrinogen**	FGA and FGB (fibrinogen beta chain): Cleaved by the protease thrombin to yield monomers, which, providing FGA and FGB are associated with fibrinogen gamma (FGG), polymerize to form an insoluble fibrin matrix. Fibrin has a major function in hemostasis as one of the primary components of blood clots. An additional function of fibrin, during the early stages of wound repair, is to stabilize the lesion and guide cell migration during re-epithelialization. Originally thought to be essential for platelet aggregation, based on in vitro studies using anticoagulated blood.
**IL4**	Interleukin-4: Participates in at least several B-cell activation processes, as well as processes in other cell types. A costimulator of DNA synthesis. Induces the expression of class II MHC molecules on resting B-cells. Enhances both secretion and cell surface expression of IgE and IgG1. Regulates the expression of the low affinity Fc receptor for IgE (CD23) on both lymphocytes and monocytes. Positively regulates IL31RA expression in macrophages.
**IL6**	Interleukin-6: A cytokine with a wide variety of biological functions. A potent inducer of the acute phase response, and plays an essential role in the final differentiation of B-cells into Ig-secreting cells. Involved in lymphocyte and monocyte differentiation. Acts on B-cells, T-cells, hepatocytes, hematopoietic progenitor cells, and cells of the central nervous system. Required for the generation of T(H)17 cells.
**TNNT1**	Troponin T: The tropomyosin-binding subunit of troponin, the thin filament regulatory complex which confers calcium-sensitivity to striated muscle actomyosin ATPase activity.
**Nitric Oxide (NO)**	NOS1 (nitric oxide synthase, brain): Produces nitric oxide (NO), which is a messenger molecule with diverse functions throughout the body. In the brain and peripheral nervous system, NO displays many properties of a neurotransmitter. Probably possesses nitrosylase activity and mediates cysteine S-nitrosylation of cytoplasmic target proteins, such as SRR; Belongs to the NOS family;<br>NOS2 (nitric oxide synthase, inducible): Produces nitric oxide (NO), which is a messenger molecule with diverse functions throughout the body. In macrophages, NO mediates tumoricidal and bactericidal activity. Possesses nitrosylase activity and mediates cysteine S-nitrosylation of cytoplasmic target proteins, such as PTGS2/COX_2;

	NOS3 (nitric oxide synthase, endothelial: Produces nitric oxide (NO), which is implicated in vascular smooth muscle relaxation through a cGMP-mediated signal transduction pathway. NO mediates vascular endothelial growth factor (VEGF)-induced angiogenesis in coronary vessels and promotes blood clotting through the activation of platelets.
**Dimethylarginine**	TDRD3 (tudor domain-containing protein 3): Scaffolding protein that specifically recognizes and binds dimethylarginine-containing proteins. In the nucleus, acts as a coactivator: recognizes and binds asymmetric dimethylates on the core histone tails associated with transcriptional activation (H3R17me2a and H4R3me2a) and recruits proteins at these arginine-methylated loci. In the cytoplasm, may play a role in the assembly and/or disassembly of mRNA stress granules, and in the regulation of translation of target mRNAs by binding Arg/Gly-rich motifs (GAR) in dimethylarginine-containing proteins;<br>AGXT2 (Alanine-glyoxylate aminotransferase 2, mitochondrial): It can metabolize asymmetric dimethylarginine (ADMA) via transamination into alpha-keto-delta-(NN-dimethylguanidino) valeric acid (DMGV). ADMA is a potent inhibitor of nitric oxide (NO) synthase, and this activity provides the mechanism through which the kidney regulates blood pressure. Belongs to the class III pyridoxal-phosphate-dependent aminotransferase family.
**Albumin**	ALB (serum albumin): the main protein of plasma, has a good binding capacity for water, $Ca^{(2+)}$, $Na^{(+)}$, $K^{(+)}$, fatty acids, hormones, bilirubin, and drugs. Main function is the regulation of the colloidal osmotic pressure of blood. Major zinc transporter in plasma, typically binds approximately 80% of all plasma zinc. Belongs to the ALB/AFP/VDB family.
**Osteocalcin**	BGLAP (osteocalcin): Constitutes 1–2% of total bone protein. Binds strongly to apatite and calcium; contains Gla domains.
**Cystatin C**	CST3 (cystatin C): As an inhibitor of cysteine proteinases, this protein is thought to serve an important physiological role as a local regulator of this enzyme's activity. Belongs to the cystatin family.
**PGLYRP1**	Peptidoglycan recognition protein 1: Pattern receptor that binds to murein peptidoglycans (PGN) of Gram-positive bacteria. Possesses bactericidal activity towards Gram-positive bacteria. May kill Gram-positive bacteria by interfering with peptidoglycan biosynthesis. Binds to Gram-negative bacteria and possesses bacteriostatic activity towards Gram-negative bacteria. Plays a role in innate immunity.
**Cholesterol**	NPC1 (Niemann–Pick C1 protein): Intracellular cholesterol transporter that acts in concert with NPC2 and plays an important role in the egress of cholesterol from the endosomal/lysosomal compartment. Both NPC1 and NPC2 function as the cellular 'tag team duo' (TTD) to catalyze the mobilization of cholesterol within the multivesicular environment of the late endosome (LE), and thus effect egress through the limiting bilayer of the LE. NPC2 binds unesterified cholesterol that has been released from LDLs in the lumen of the late endosomes/lysosomes;<br>CYP7A1 (cholesterol 7-alpha-monooxygenase): Catalyzes a rate-limiting step in cholesterol catabolism and bile acid biosynthesis by introducing a hydrophilic moiety at position 7 of cholesterol. Important for cholesterol homeostasis. Belongs to the cytochrome P450 7 family.
**HDL**	HSD11B1 (corticosteroid 11-beta-dehydrogenase isozyme 1): Catalyzes, reversibly, the conversion of cortisol to the inactive metabolite cortisone. Catalyzes, reversibly, the conversion of 7-ketocholesterol to 7-beta-hydroxycholesterol. In intact cells, the reaction runs only in one direction, from 7-ketocholesterol to 7-beta-hydroxycholesterol. Belongs to the short-chain dehydrogenase/reductase superfamily;<br>SCARB1 (scavenger receptor class B member 1): Receptor for different ligands, such as phospholipids, cholesterol ester, lipoproteins, phosphatidylserine, and apoptotic cells. Receptor for HDL, mediating selective uptake of cholesteryl ether and HDL-dependent cholesterol efflux. Facilitates the flux of free and esterified cholesterol between the cell surface and apoB-containing lipoproteins and modified lipoproteins, although less efficiently than HDL. May be involved in the phagocytosis of apoptotic cells via its phosphatidylserine binding activity.
**LDL**	PON2 (serum paraoxonase/arylesterase 2): Capable of hydrolyzing lactones and a number of aromatic carboxylic acid esters. Possesses antioxidant activity. Not associated with high-density lipoproteins. Prevents LDL lipid peroxidation, reverses the oxidation of mildly oxidized LDL, and inhibits the ability of MM-LDL to induce monocyte chemotaxis;<br>LDLRAP1 (low-density lipoprotein receptor adapter protein 1): Adapter protein (clathrin-associated sorting protein (CLASP)) required for efficient endocytosis of the LDL receptor (LDLR) in polarized cells, such as hepatocytes and lymphocytes, but not in non-polarized cells (fibroblasts). May be required for LDL binding and internalization, but not for receptor clustering in coated pits. May facilitate the endocytocis of LDLR and LDLR-LDL complexes from coated pits by stabilizing the interaction between the receptor and the structural components of the pits;

	APOB (apolipoprotein B-100): Apolipoprotein B is a major protein constituent of chylomicrons (apo B-48), LDL (apo B-100), and VLDL (apo B-100). Apo B-100 functions as a recognition signal for the cellular binding and internalization of LDL particles by the apo B/E receptor.
**Triglycerides**	CETP (cholesteryl ester transfer protein): Involved in the transfer of neutral lipids, including cholesteryl ester and triglyceride, among lipoprotein particles. Allows the net movement of cholesteryl ester from high-density lipoproteins (HDL) to triglyceride-rich very low-density lipoproteins (VLDL), and the equimolar transport of triglyceride from VLDL to HDL. Regulates reverse cholesterol transport, by which excess cholesterol is removed from peripheral tissues and returned to the liver for elimination. Belongs to the BPI/LBP/Plunc superfamily;<br>APOC3 (apolipoprotein C-III): Component of triglyceride-rich very low-density lipoproteins (VLDL) and high-density lipoproteins (HDL) in plasma. Plays a multifaceted role in triglyceride homeostasis. Intracellularly promotes hepatic very low-density lipoprotein 1 (VLDL1) assembly and secretion; extracellularly attenuates hydrolysis and clearance of triglyceride-rich lipoproteins (TRLs). Impairs the lipolysis of TRLs by inhibiting lipoprotein lipase and the hepatic uptake of TRLs by remnant receptors.
**Hemoglobin**	AHSP (alpha-hemoglobin-stabilizing protein): Acts as a chaperone to prevent the harmful aggregation of alpha-hemoglobin during normal erythroid cell development. Specifically protects free alpha-hemoglobin from precipitation. Predicted to modulate pathological states of alpha-hemoglobin excess, such as beta-thalassemia. Belongs to the AHSP family;<br>HBA2 (hemoglobin subunit alpha 2): Involved in oxygen transport from the lungs to the various peripheral tissues;<br>HBZ (Hemoglobin subunit zeta): The zeta chain is an alpha-type chain of mammalian embryonic hemoglobin.

Source: string-db.org/ (Accessed on 27 January 2021).

Supplementary Table S2. Biomarkers analyzed in the included studies.

Author (year)	CRP	Albumin	Calcium	Phosphorus	Potassium	Magnesium	Urea	Creatinine	Ferritin	Fibrinogen	Triglyceride	Osteocalcin	Iron	Fasting blood glucose	LDL	HDL	Uric acid	Total cholesterol	Transferrin
**Cotič *et al.* 2017**	6.1<br>(0.2-241)	N/A	N/A	N/A	N/A	N/A	N/A	N/A	N/A	N/A	N/A	N/A	N/A	N/A	N/A	N/A	N/A	N/A	N/A
**Hou *et al.* 2017**	PD:4.66±1.84<br><br>No PD:<br>2.62±1.20<br>(*p*:<0.001)	N/A	PD;2.07±0.23<br><br>No PD:<br>2.11±0.22<br>(*p*=.380)	PD:1.66±0.48<br><br>No PD:<br>2.29±3.82<br>(*p*=.174)	PD:4.71±0.78<br><br>No PD:<br>4.73±0.60<br>(*p*=.877)	N/A	N/A	N/A	N/A	N/A	PD:1.56±0.52<br><br>No<br>PD:1.49±0.56<br>(*p*=.442)	N/A	N/A	PD:8.04±2.17<br><br>No<br>PD:5.76±1.51<br>(*p*<0.001)	PD:2.71±0.64<br><br>No<br>PD:2.51±1.14<br>(*p*=.215)	PD:1.16±0.33<br><br>No<br>PD:1.20±0.37<br>(*p*=.557)	N/A	N/A	N/A
**Veisa *et al.* 2017**	9.29 ± 15.21	4.29 ± 0.42	N/A	N/A	N/A	N/A	N/A	N/A	942.57 ± 712.75	N/A	N/A	N/Av	N/A	N/A	N/A	N/A	N/A	N/A	N/A
**Garneat *et al.* 2014**	Healthy:3 (1-9)<br><br>PD:5 (1-12)<br>(*p*=.252)	Healthy:4.1 ± 6 0.4<br><br>PD:4.1 ± 6<br>0.2 (*p*=.235)	N/A	N/A	N/A	N/A	N/A	N/A	N/A	N/A	N/A	N/A	N/A	N/A	N/A	N/A	N/A	N/A	N/A
**Ksiazek *et al.* 2019**	N/A	N/A	N/A	N/A	N/A	N/A	N/A	N/A	N/A	N/A	N/A	N/A	N/A	N/A	N/A	N/A	N/A	N/A	N/A
**Perozini *et al.* 2017**	EG: 3.4 ± 3.4<br><br>PDG:4.6 ±<br>3.5<br><br>HDG:3.5 ±1.3<br>(*p*=.1190)	N/A	N/A	N/A	N/A	N/A	N/A	N/A	EG:134.3± 39.6<br><br>PDG:218.1 ± 145.8<br><br>HDG:1004.0±952.1<br>(*p*<0.0001)	EG:151.5 ±<br>67.3<br><br>PDG:167.7 ±<br>55.2<br><br>HDG:206.9 ±<br>48.3<br>(*p*<0.0001)	EG:151.5± 67.3<br><br>PDG:167.7 ±<br>55.2<br><br>HDG:206.9 ±<br>48.3<br>(*p*<0.0001)	N/A	N/A	N/A	N/A	N/A	N/A	N/A	N/A
**Yoshihara *et al.* 2016**	N/A	N/A	N/A	N/A	N/A	N/A	N/A	N/A	N/A	N/A	N/A	Worst PD with higher levels of osteocalcin	N/A	N/A	N/A	N/A	N/A	N/A	N/A
**Rodrigues *et al.* 2014**	N/A	PD:3.44 ±<br>0.44<br><br>No PD: 3.78±<br>0.31 (*p*=.021)	PD:8.67±0.42<br><br>No PD:8.68 ±<br>0.39<br>(*p*=.957)	PD:5.02±1.19<br><br>No PD:<br>6.25±1.72<br>(*p*=.024)	PD:4.65± 0.61<br><br>No PD:4.94 ±<br>0.72<br>(*p*=.220)	N/A	N/A	PD: 10.05 ±<br>4.71<br><br>No PD: 9.76<br>±4.82<br>(*p*=.864)	PD: 227.7 ± 76.7<br><br>No PD: 306.3±142.3<br>(*p*=.185)	N/A	N/A	N/A	PD:74.17<br>±62.15<br><br>No PD:<br>93.77<br>±70.17<br>(*p*=.409)	N/A	N/A	N/A	N/A	N/A	PD:211.7 ±<br>68.2<br><br>No PD:<br>263.8 ±81.4<br>(*p*=.058)
**Chen *et al.* 2015**	N/A	4.35 6 0.32<br>4.32 6 0.32<br>*(p=.001)*	N/A	N/A	N/A	N/A	18.1 6<br>7.3 17.7<br>6 6.6<br>,0.001	N/A	N/A	N/A	127.8 6 76.0<br>126.4 6 78.4<br>(0.05)	N/A	N/A	N/A	N/A	52.4 6 14.5 53.2<br>6 14.3 *(p=.001)*	6.1 6 1.6<br>5.9 6 1.6<br>*(p=.001)*	PD:195.7± 6<br>36.6<br><br>No PD: 197.5<br>±6 36.5<br>(*p*=.001)	N/A
**Grubbs *et al.* 2019**	CRP decreased	N/A	N/A	N/A	N/A	N/A	N/A	N/A	N/A	N/A	N/A	N/A	N/A	N/A	N/A	N/A	N/A	N/A	N/A
**Arenious *et al.* 2020**	Hemodialysis patients with a higher S. aureus, which is associated with high PGLYRP1	N/A	N/A	N/A	N/A	N/A	N/A	N/A	N/A	N/A	N/A	N/A	N/A	N/A	N/A	N/A	N/A	N/A	N/A
**Ausavarungnirun *et al.* 2016**	N/A	Decreased in worst CKD	N/A	N/A	N/A	N/A	N/A	N/A	N/A	N/A	N/A	N/A	N/A	N/A	N/A	N/A	N/A	N/A	N/A

N/A = Not analyzed/quantified; CRP = C-reactive protein; PD = With periodontal disease; EG = Early-stage group; HDG = Hemodialysis group; PDG = Predialysis group; PGLYRP1 = Peptidoglycan Recognition Protein 1.
